# Supplementary material for: Impact of electronic immunization registries and electronic logistics management information systems in four low-and middle-income countries: Guinea, Honduras, Rwanda, and Tanzania
Source: Vaccine. 2025 Apr 30;54:None. doi: 10.1016/j.vaccine.2025.127066 (PMC12132044; doi:10.1016/j.vaccine.2025.127066)
Supplement: Supplementary file 1 — Supplementary material 1 [file mmc1.pdf]

District/Regional Interview Guide

DISTRICT/REGION INTERVIEW GUIDE (for all districts/regions)

Date of interview

yyyy-mm-dd

Name of interviewer

Province / Region

- ◯ Mbeya
- ◯ Arusha
- ◯ Dodoma
- ◯ Njombe
- ◯ Mwanza
- ◯ Kilimanjaro
- ◯ Tanga
- ◯ Singida
- ◯ Shinyanga
- ◯ Pwani

District

Role within immunization service

1

* **Name of interviewee**

Role within immunization services

- ◯ Vaccine Officer
- ◯ Cold Chain Officer
- ◯ DIVO
- ◯ RIVO
- ◯ Registered nurse
- ◯ Data clerk
- ◯ Other

* **Other, specify**

A. Background

How often in the last month have you used VIMS/eIS?

- ◯ Every day
- ◯ A few times a week
- ◯ Once a month
- ◯ A few times a month
- ◯ Not at all

Is your immunization system set-up [paper based or electronic (VIMS/eIS)] or do you use both systems?

- ◯ VIMS + Paper-based Immunization register
- ◯ eIS: VIMS+TImR
- ◯ Both in parallel eIS (VIMS + eIS) + paper-based immunization register

Please describe how you use the system?

Tick all appropriate boxes and explain.

- ☐ Decision making
- ☐ Planning
- ☐ Reporting to higher levels
- ☐ Providing feedback to lower levels
- ☐ Information gathering
- ☐ Budgeting
- ☐ Other

Other, please specify

____

Explain and provide examples

____

For VIMS/eIS: Have there been any challenges that prevented you from using the VIMS/eIS?

- ☐ No electricity
- ☐ No internet connectivity
- ☐ No data bundles
- ☐ Not enough computers laptops tablets
- ☐ Broken computer laptops tablets
- ☐ Other

Other, please specify

____

Have you had an Adverse Event Following Immunization (AEFI) reported from any of your facilities in the past year?

- Yes
- No
- Not sure

For VIMS/eIS: If yes, did you use the VIMS/eIS to report the AEFI?

- Yes
- No
- Not sure

Please provide details

What is your current process for defaulter tracking?

Interviewer: when EIS is present, probe for the use of the EIS in defaulter tracking

---

Does the system help track individuals receiving vaccines outside their catchment areas?

- Yes
- No

If yes, please describe, include whether use of paper-based or electronic system

---

B. Denominators

Is a monthly target population available for the number of children under 1 year for the District/Region?

- Yes
- No

If yes, what is the source of this target population estimate?

What is the monthly target population of children under 1 year?

Do you think the target population is accurate (i.e., is it reflective of the true catchment population for this health centre)?

- Yes
- No

If no, why not?

- True catchment population is larger
- True catchment population is smaller

C. Infrastructure

Is there a computer /laptop/tablet available for immunization activities?

- Yes
- No

Is this computer/tablet/laptop for use exclusively for immunization program activities?

- Yes
- No

If no, what else is it used for?

Is there a printer available for immunization services?

- Yes
- No

How many times in the past week have you not been able to charge the computer/laptop/tablet due to lack of electricity?

- I could charge it and use it every day.
- Once
- Two – Four times
- I have not been able to use it this week as we haven't had electricity
- Other

Other, please specify:

How many times in the past week have you not been able to send data due to lack of electricity/ internet connectivity?

- I could send data whenever I needed to
- Once
- Three – Four times
- I haven't been able to send data this week as we haven't had electricity/connectivity/data bundles
- Other

Other, please specify:

Do you think you have enough computers/laptops/tablets/mobile phones for your storage facility?

- ◯ Yes
- ◯ No
- ◯ Not sure
- ◯ Other

Other, please specify:

In the past year, have you had any computers/laptops/tablets/mobile phones stop working?

- ◯ Not often
- ◯ Never
- ◯ Other

Other, please specify:

How long does it take a broken computer/laptop/tablet to get fixed or replaced?

- ◯ A day
- ◯ A week
- ◯ A month
- ◯ More than a month
- ◯ Unsure

Please describe the process to get it fixed.

Do you have internet connection?

- ◯ Yes
- ◯ No

What kind of connection do you have?

- ☐ Mobile data bundles
- ☐ Fibre/cable
- ☐ Satellite
- ☐ Other

Other, specify:

What type of electricity do you have (tick all relevant)

- [ ] Government Grid
- [ ] Generator only
- [ ] Solar power
- [ ] Other

Other, specify:

Does your district/region experience power outages?

- ( ) Yes
- ( ) No

If yes, is there a backup source?

- ( ) Yes
- ( ) No

Specify backup

Overview

I think the electronic system (VIMS/eIS) is user friendly

Interviewer: this is perception (first reaction) based on the tool being simple, clear, intuitive and reliable

- ( ) Yes
- ( ) No
- ( ) Not sure

Overall, I think that the electronic system (VIMS/eIS) is functioning when I need to use it

- ( ) Yes
- ( ) No
- ( ) Not sure

D. Vaccine Management

Describe how you manage vaccine stock.

Interviewer: Take note of the different tools (paper and electronic) used

---

Has your (District/Regional) vaccine store experienced any vaccine stock-outs in the last 3 months?

- ◯ Yes
- ◯ No

If yes, describe the reason

(e.g., higher-than expected consumptions, expiry/wastage, late delivery, lack of replenishment from supplying store, other)

If yes, which vaccines were out of stock?

- ☐ Pentavalent
- ☐ PCV
- ☐ HPV
- ☐ MR
- ☐ Other

Other, please specify:

What was the longest amount of time the vaccines were out of stock?

- ◯ Less than 1 week
- ◯ 1-2 weeks
- ◯ 3-4 weeks
- ◯ 1 month or more

What is your monthly requirement for Pentavalent vaccine?

Add the number

Is your current stock balance for Pentavalent vaccine

(Perform personal check)

- ◯ Above maximum stock (6 weeks or more)
- ◯ Appropriate stock level
- ◯ Below minimum stock (1 week or less)
- ◯ Stocked-out

Comment on the stock balance described above

Does having the VIMS /eIS allow you to manage stock more effectively or efficiently than before?

- ◯ Yes, it is better using the electronic tool
- ◯ No, it was better using the paper tool
- ◯ Not sure

Explain and provide examples where possible

E. Data Quality

Do you feel that the vaccine stock data quality in the VIMS/eIS is:

- Much worse than the paper stock card/ledger
- A little bit worse than the paper stock card/ledger
- About the same as the paper stock card/ledger
- A little bit better than the paper stock card/ledger
- Much better than the paper stock card/ledger

For VIMS/eIS: Do you feel that the immunization programme data quality in the VIMS/eIS is:

- Much worse than the paper stock card/ledger
- A little bit worse than the paper stock card/ledger
- About the same as the paper stock card/ledger
- A little bit better than the paper stock card/ledger
- Much better than the paper stock card/ledger

For VIMS/eIS: Do you feel that AEFI data quality in the VIMS/eIS is:

- Much worse than the paper registry
- A little bit worse than the paper registry
- About the same as the paper registry
- A little bit better than the paper registry
- Much better than the paper registry

What do you think is the most accurate source of data for an individual child's immunization history?
(Interviewer: this is opinion/perception of the respondent)

- Immunization registry (paper)
- eIS (electronic)
- Child Health Card (RCH Card 1)
- Caregiver recall
- Other

Other, please specify:

What are the most common challenges with getting correct immunization and vaccine stock data in your (District/Regional) office? Please explain.

Do you conduct data quality audits at the District/Regional office?

- Yes
- No
- Not sure
- Other

Other, please specify:

Is it for only immunization data or all health programme data

- Immunization only
- Immunization and other programmes combined

If yes, how often do you conduct such data quality audits?

- Once a month
- Once a quarter
- Once a year
- Once every 3 – 5 years
- Not sure
- Other

Other, please specify:

If no, how do you manage your data quality?

Do you produce a report on the data quality audit?

- Yes
- No
- Not sure

Please Explain

Do you have a data improvement plan for your district/region?

- Yes
- No
- Not sure

If yes, is it funded?

- Yes
- No
- Not sure

F. Data analysis, Interpretation and Use

Is there an immunization performance monitoring chart, dashboard, or other means of data visualisation (maps) available at the (District/Region)?

- Yes
- No

If yes, is the performance monitoring chart, dashboard etc. up to date (updated to last complete month)?

- Yes
- No
- Partly

If yes, is the performance monitoring chart / dashboard electronic or in paper-format?

- Electronic
- Paper
- Both

If no, how is immunization data monitored (please describe)

Can the (District/Region) identify the three health facilities with the lowest Penta3, MR2, PCV3, and HPV2 coverage rates for the last three months?

- Yes
- No

If yes, briefly explain the reasons for these low coverage rates

If no, comment why not

Do you have a (District/Region)-immunization microplan?

Interviewer:probe about use of paper-based or electronic tools in developing microplan

- Yes
- No

If so, briefly explain what data are used for developing the plan

______

Does the (District/Region) prioritize the needs of health facilities based on available performance data?

- ◯ Yes
- ◯ No

If yes, what indicators are used to prioritize health facilities by performance?

______

What is the source of these performance data?

- ◯ Paper-based data system
- ◯ VIMS
- ◯ TImR

How are the VIMS or EIS data used for decision making within the immunization program?

- ☐ Funding requests
- ☐ Request for new staff
- ☐ Forecasting vaccine needs
- ☐ Share with community
- ☐ Supervision activities
- ☐ Determine training needs
- ☐ Quality improvement efforts
- ☐ Planning
- ☐ Prioritize commodities
- ☐ Other

Other, please specify:

______

Can you give me an example of decisions you have made using data from the VIMS/eIS?

______

Reviewing your immunization and vaccine data, are there any issues or challenges you have identified that you would like to address?

- ◯ Yes
- ◯ No
- ◯ Not sure
- ◯ Other

Other, please specify:

______

If yes, what are those challenges (on data quality and use)?

What would you like to do to address these challenges?

Is there anything that prevents you from following through on the solution?

Is there a regular meeting to review and discuss immunization, vaccine stock and cold chain data and results among District/Regional immunization staff?

- ◯ Yes
- ◯ No

Describe how decisions are made about how best to manage District/Regional immunization services and vaccine management are made? Are you using immunization data to make changes?

For example, to change the days of vaccination services, do more outreach activities, support tracking of missed children, change the amount of stock you order or receive, change the frequency of receiving new supplies, or transfer stock to another facility? Probe for use of electronic tool

Based on your experience, please rate the immunization services and vaccine management decisions taken before the implementation of the VIMS or eIS, on a scale from 1-5 (1=poor, 5=excellent).

Rating from 1-5

Based on your experience, please rate the immunization services and vaccine management decisions taken currently with use of the VIMS or eIS, on a scale from 1-5 (1=poor, 5=excellent).

Rating from 1-5

G. Reporting

Ask to see the last 6 monthly monthsimmunization monthly reports submitted to the (District/Regional) office. Did the District/Regional office submit ALL of the last 6 monthly reports?

- ◯ Yes, (100% of reports submitted)
- ◯ No, (<100% of reports submitted)
- ◯ Not found

Did the (District/Regional) office submit ALL of their last 6 monthly stock management reports on time

- ◯ Yes, (100% of reports on-time)
- ◯ No, (<100% of reports on-time)
- ◯ Not found

Does the District/Regional office have a system of archiving or storing paper records?

- Yes
- No
- Not sure

Do you receive feedback from your supervisors regarding your immunization and vaccine stock data reports?

- Yes
- No
- Not sure

If yes, provide details.

If yes, how often does this happen?

- Once a month
- Once a quarter
- Once a year
- Other

Other, specify

If yes, what topics are typically covered in such a feedback?

If yes, how is this feedback provided?

- Written (electronic)
- Written (paper)
- In a face to face meeting
- Over the phone
- Informally
- Other

Other, specify

If yes, has the VIMS or eIS made this process of providing feedback easier?

- Yes
- No
- Not sure

Please explain and provide examples:

Do you provide feedback to the health facilities regarding their reported immunization and vaccine stock data?

- Yes
- No
- Not sure

Please explain:

If yes, how often does this happen?

- Once a month
- Once a quarter
- Once a year
- Other

Other, specify

If yes, what topics are typically covered in such a feedback?

If yes, how is this feedback provided?

- Written (electronic)
- Written (paper)
- In a face to face meeting
- Over the phone
- Informally
- Other

Other, specify

If yes, has the VIMS or eIS made this process of providing feedback easier?

- Yes
- No
- Not sure

Please explain and provide examples:

Please describe what electronic reports you use and if these meet your needs? If these reports do not meet your needs, what could be added or changed?

Approximately how long does/did it take to complete the receiving and put-away of new vaccine supplies using paper forms (delivery notes and stock cards/registers/ledgers?)

Interviewer: Respondent will have to rely on their memory to estimate how long it use to take using paper forms (before the introduction of VIMS).

Approximately how long does/did it take to complete the receiving and put-away of new vaccine supplies using the electronic tool (VIMS)?

minutes

Since the introduction of VIMS, is it:

- ◯ Faster using VIMS
- ◯ Slower using VIMS (it was faster using paper forms)
- ◯ No change
- ◯ Not sure

Other, please specify:

Please explain why.

H. Workforce and Training

How many staff are working in the (District/Region) immunization office and vaccine store, currently?

(Number)

What was the impact of the eIS on staff management (hiring of additional personnel, reorganization of responsibilities, shrinking/enlarging of team)?

Training and Supervision

Of the (District/Region) level staff working in immunization, how many are trained on immunization and vaccine stock data management activities at the (District/Regional) office ?

(Interviewer: probe with examples of activities such as completing or reviewing monthly immunization coverage reports, analysing vaccine stock status to avoid stock imbalances, etc.).

For VIMS/eIS: Do you think that you are adequately trained on the electronic tools in order to properly do your work responsibilities?

- ◯ Yes
- ◯ No

For paper tool: Do you think that you are adequately trained on the paper based tools in order to properly do your work responsibilities?

- ◯ Yes
- ◯ No

If no please explain

For VIMS/eIS: What supporting /training tools for VIMS/eIS do you have for your use at the District/Regional office?

- ☐ Instruction manuals
- ☐ Job aids/quick reference sheets
- ☐ Refresher trainings

Please provide details

What supporting /training tools for paper based tools do you have for your use at the facility?

- ☐ Instruction manuals
- ☐ Job aids/quick reference sheets
- ☐ Refresher trainings

Please provide details

If relevant, when were these supporting/training materials (for electronic tools) last updated or reviewed?

- ◯ Never
- ◯ When a change was made to the system
- ◯ They don't need to be updated
- ◯ They need to be updated

If relevant, when were these supporting/training materials (for paper based tools) last updated or reviewed?

- ◯ Never
- ◯ When a change was made to the system
- ◯ They don't need to be updated
- ◯ They need to be updated

Please provide details

For VIMS/eIS: Do you clearly understand your role and responsibilities in using the VIMS/eIS?

- ○ No, it is not clear
- ○ I understand my role and responsibilities but I am not properly equipped
- ○ I understand my role and responsibilities and I am well equipped to carry out my responsibilities

How many immunization supervision activities has the higher level conducted with this District/Region in the past year looking specifically at immunization programme and vaccine logistics data?

- ○ No supervision
- ○ Once least once a year
- ○ Once a quarter
- ○ Once a month
- ○ Other

Other, please specify

___

For VIMS/eIS: If one or more immunization supervision activities were conducted, did you use the VIMS/eIS or data from the VIMS/eIS to guide your discussion?

- ○ Yes
- ○ No

If yes, please describe how the data was used?

___

Are there specific items regarding the VIMS/ eIS that you usually cover during supportive supervision visits? Please describe:

___

I. Close and Comments

If another health district/region was considering using the VIMS/eIS for their immunization records and vaccine logistics management and asked you for advice, what would you tell them about benefits, challenges and best practices?

___

What electronic tools are you using to manage COVID-19 vaccine delivery e.g., for stock management and/or for individual vaccination?

___

Final notes and comments:

___
