# Supplementary material for: Impact of electronic immunization registries and electronic logistics management information systems in four low-and middle-income countries: Guinea, Honduras, Rwanda, and Tanzania
Source: Vaccine. 2025 Apr 30;54:None. doi: 10.1016/j.vaccine.2025.127066 (PMC12132044; doi:10.1016/j.vaccine.2025.127066)
Supplement: Supplementary file 2 — Supplementary material 2 [file mmc2.pdf]

Caregiver Interview guide (only for facilities where TImR exists)

Only for facilities where TImR exists

Is TImR available at the facility?

- Yes
- No

This survey is not required (only for facilities where TImR exists).

Date of interview

yyyy-mm-dd

Name of interviewer

Province / Region

- Mbeya
- Njombe
- Tanga
- Kilimanjaro
- Arusha
- Dodoma
- Singida
- Shinyanga
- Mwanza
- Pwani

District

Health facility name

Is this the first or second health facility visited in the district?

- First facility visited
- Second facility visited

Gender

- Male
- Female

Role

- Mother/Father
- Family member
- Other

Others specify

This questionnaire should be administered to individuals after they complete vaccination at the facility/site.

Note to interviewer: Please use the script to approach potential interviewees to explain the purpose of the interview and request their participation.

Begin the interview by saying the following: "I would like to ask you a few questions about your experience getting the child vaccinated. The answers you give will help us learn more about how to improve immunization service delivery. This survey is completely voluntary, and your answers will be completely anonymous. You may withdraw at any time and stop participation without penalty".

Did you notice if the staff used an electronic tool, like a tablet or smartphone today to record your visit?

- Yes
- No
- Not sure

Have you noticed any difference to your visits to the clinics to get the child's vaccines since the staff have started using the electronic tool?

- Yes
- No
- Not sure

How do you think the electronic tool use has impacted your visit?

interviewer: tick as many boxes that apply but do not read out the list

- [ ] Waiting times are more
- [ ] Waiting times are less
- [ ] I don't have to bring my clinic card anymore
- [ ] The facility is more organised
- [ ] The facility is more disorganised
- [ ] It is worse than when they just used the paper tools
- [ ] It is better than when they just used the paper tools
- [ ] Other

Others specify

Please explain your responses. Why is this so?

Has your child missed/been late for any vaccine doses?

- Yes, but s/he is now up to date
- Yes, but s/he is still not up to date
- No
- Not, sure
- Other

Others specify

If yes, why? Could you please describe why your child was late?

- I forgot
- I didn't have the time to come here
- There was no vaccine
- COVID-19 restrictions
- I didn't know I should bring the child
- Other

Others specify

If the child had missed/been late for any doses, did you receive a reminder to come for the child's vaccine?

- Yes
- No
- NA

If yes by:

- [ ] SMS
- [ ] Phone call
- [ ] Visit from a Community Health Worker
- [ ] Other

Others specify
