# Supplementary material for: Impact of electronic immunization registries and electronic logistics management information systems in four low-and middle-income countries: Guinea, Honduras, Rwanda, and Tanzania
Source: Vaccine. 2025 Apr 30;54:None. doi: 10.1016/j.vaccine.2025.127066 (PMC12132044; doi:10.1016/j.vaccine.2025.127066)
Supplement: Supplementary file 3 — Supplementary material 3 [file mmc3.pdf]

Facility competency assessment

General Information

Date of assessment

yyyy-mm-dd

Name of interviewer

Province / Region

- ◯ Mbeya
- ◯ Njombe
- ◯ Tanga
- ◯ Kilimanjaro
- ◯ Arusha
- ◯ Dodoma
- ◯ Singida
- ◯ Shinyanga
- ◯ Mwanza
- ◯ Pwani

District

Health facility name

Is this the first or second health facility visited in the district?

- ◯ First facility visited
- ◯ Second facility visited

Location of the facility

- ◯ Urban
- ◯ Rural/Remote
- ◯ Suburban
- ◯ Other

Level of health facility

- ◯ Hospital
- ◯ Health centre
- ◯ Dispensary
- ◯ Clinic

Type of health facility

- ◯ Public
- ◯ Private
- ◯ Other

other
enter only when others is selected

Role within immunization service

1

* **Name of interviewee**

Role within immunization services

- ◯ Head of clinic
- ◯ Vaccinator
- ◯ Data capture
- ◯ Other

* **others specify**
enter only when others is selected

Is TImR available at the facility?

- ◯ Yes
- ◯ No

When was the TImR implemented?

yyyy-mm-dd

Instructions

For these assessments, the competency criteria are defined as follows: .

• Fully competent = completes whole task accurately/correctly, confidently (without hesitation/consultation) + rapidly

---

• Mostly competent = mostly completes task accurately/correctly, confidently (without hesitation/consultation) + rapidly

---

• Some competence = only partly completes task accurately/correctly, not fully confident (some hesitation/consultation) + somewhat slow

---

• Little/no competence = cannot complete task or does so incorrectly, with significant hesitation and/or takes a long time (>5 minutes)

Can you please demonstrate to me how you use the TImR:
Interviewer: This is for you to get an overview of how the tool is used. You can note general observations.

---

1. Can the health worker assessed demonstrate how to complete a new TImR immunization record (including saving and uploading data)?

Fully competent user 1

- ○ Yes
- ○ No

Fully competent user 2

- ○ Yes
- ○ No

Mostly competent user 1

- ○ Yes
- ○ No

Mostly competent user 2

- ○ Yes
- ○ No

Some competence user 1

- Yes
- No

Some competence user 2

- Yes
- No

Little/no competence 1

- Yes
- No

Little/no competence 2

- Yes
- No

Notes

---

2. Can the health workers assessed demonstrate how to use the tablet to generate an immunization status report for the facility?

Fully competent user 1

- Yes
- No

Fully competent user 2

- Yes
- No

Mostly competent user 1

- Yes
- No

Mostly competent user 2

- Yes
- No

Some competence user 1

- Yes
- No

Some competence user 2

- Yes
- No

Little/no competence 1

- Yes
- No

Little/no competence 2

- Yes
- No

Notes

3. Can the health workers assessed demonstrate how to correctly interpret the immunization status report? (including appropriate actions)

Fully competent user 1

- Yes
- No

Fully competent user 2

- Yes
- No

Mostly competent user 1

- Yes
- No

Mostly competent user 2

- Yes
- No

Some competence user 1

- Yes
- No

Some competence user 2

- Yes
- No

Little/no competence 1

- Yes
- No

Little/no competence 2

- Yes
- No

Notes

---

4. Can the health workers assessed demonstrate how to use the tablet to generate a report on defaulters?

Fully competent user 1

- Yes
- No

Fully competent user 2

- Yes
- No

Mostly competent user 1

- Yes
- No

Mostly competent user 2

- Yes
- No

Some competence user 1

- Yes
- No

Some competence user 2

- Yes
- No

Little/no competence 1

- Yes
- No

Little/no competence 2

- Yes
- No

Notes

---

5. Can the health workers assessed demonstrate how to correctly interpret the defaulter report? (including appropriate actions)

Fully competent user 1

- Yes
- No

Fully competent user 2

- Yes
- No

Mostly competent user 1

- Yes
- No

Mostly competent user 2

- Yes
- No

Some competence user 1

- Yes
- No

Some competence user 2

- Yes
- No

Little/no competence 1

- Yes
- No

Little/no competence 2

- Yes
- No

Please report the actions they are describing:

Notes

6. Can the health workers assessed demonstrate how to use the tablet to generate a report on vaccine stock?

Fully competent user 1

- Yes
- No

Fully competent user 2

- Yes
- No

Mostly competent user 1

- Yes
- No

Mostly competent user 2

- Yes
- No

Some competence user 1

- Yes
- No

Some competence user 2

- Yes
- No

Little/no competence 1

- Yes
- No

Little/no competence 2

- Yes
- No

Notes

---

7. Can the health workers assessed demonstrate how to confirm receipt of new vaccine stock?

Fully competent user 1

- Yes
- No

Fully competent user 2

- Yes
- No

Mostly competent user 1

- Yes
- No

Mostly competent user 2

- Yes
- No

Some competence user 1

- Yes
- No

Some competence user 2

- Yes
- No

Little/no competence 1

- Yes
- No

Little/no competence 2

- Yes
- No

Notes

---

8. Can the health workers assessed demonstrate how to adjust TImR stock data for wastage?

Fully competent user 1

- Yes
- No

Fully competent user 2

- Yes
- No

Mostly competent user 1

- Yes
- No

Mostly competent user 2

- Yes
- No

Some competence user 1

- Yes
- No

Some competence user 2

- Yes
- No

Little/no competence 1

- Yes
- No

Little/no competence 2

- Yes
- No

Notes

---

9. Can the health workers assessed demonstrate how to correctly interpret a stock report to determine if they are overstocked, understocked, or at risk of product expiring? (including appropriate actions)

Fully competent user 1

- Yes
- No

Fully competent user 2

- Yes
- No

Mostly competent user 1

- Yes
- No

Mostly competent user 2

- Yes
- No

Some competence user 1

- Yes
- No

Some competence user 2

- Yes
- No

Little/no competence 1

- Yes
- No

Little/no competence 2

- Yes
- No

Notes

10. Are there any additional comments on the use of TImR/EIS?

Interviewer: Note comments/concerns verbatim.

11. Overall comments from interviewer/ observer about subject'sabilities to demonstrate competency with TImR/EIS, the phone/tablet, or the interpretation of data
