# Supplementary material for: Impact of electronic immunization registries and electronic logistics management information systems in four low-and middle-income countries: Guinea, Honduras, Rwanda, and Tanzania
Source: Vaccine. 2025 Apr 30;54:None. doi: 10.1016/j.vaccine.2025.127066 (PMC12132044; doi:10.1016/j.vaccine.2025.127066)
Supplement: Supplementary file 4 — Supplementary material 4 [file mmc4.pdf]

Facility Interview Guide (for all facilities)

Facility Interview Guide (for all facilities)

Date of interview

yyyy-mm-dd

Name of interviewer

Province / Region

- ◯ Mbeya
- ◯ Njombe
- ◯ Tanga
- ◯ Kilimanjaro
- ◯ Arusha
- ◯ Dodoma
- ◯ Singida
- ◯ Shinyanga
- ◯ Mwanza
- ◯ Pwani

District

Health facility name

Is this the first or second health facility visited in the district?

- ◯ First facility visited
- ◯ Second facility visited

Role within immunization service

1

* **Name of interviewee**

Role within immunization services

- ◯ Head of clinic
- ◯ Vaccinator
- ◯ Data capturer
- ◯ Other

* **others specify**

Enter only when others is selected

System in use

Has TImR been introduced at your facility?

Interviewer: Some facilities might be in districts with TImR but may not be using it currently. If it was introduced but not currently in use, answer 'yes' here, and/or if exists in the district/region, answer 'yes'.

- Yes
- No

When was the TImR implemented?

This can be an approximate date

yyyy-mm

Do you use both the TImR and a paper-based system for the management of your immunization information?

- TImR only
- Paper only
- TImR and paper

A. BACKGROUND

How many days a week are vaccination services provided?

Describe the practice of recording vaccine doses administered.

In which tool(s) are vaccine doses recorded?

- [ ] IVD immunization registry (paper)
- [ ] TImR (electronic)
- [ ] Child register (Book 7)
- [ ] Child health card (RCH Card 1)
- [ ] Immunization monthly report
- [ ] Immunization tally sheet
- [ ] Ledger book
- [ ] Not recorded
- [ ] Other

Other:

What do you think is the most accurate source of data for an individual child's immunization history?

Interviewer: this is opinion/perception of the respondent

- IVD immunization registry (paper)
- TImR (Electronic)
- Child health card (RCH Card 1)
- Caregiver recall
- Other

Other, please specify:

B. Defaulter tracking

Is there an immunization defaulter-tracking mechanism that uses data available at the health facility?

- Yes
- No

If yes, how is defaulter tracking done? Please describe the process.

Do you think the process is effective?

- Yes
- No

If no, explain if/how defaulters are tracked.

Does your health facility use TImR to notify caregivers:

(a) of an upcoming scheduled vaccination and/or

- Yes
- No

(b) who have missed vaccinations for their children (the vaccine is overdue)?

- Yes
- No

If not electronically, how are caregivers notified?

If yes, how effective do you think these reminders are for getting people to attend and for preventing defaulters?

- Very effective
- Quite effective
- Not effective

Please explain your response

Are you easily able to identify children in the TImR that were registered at a different health facility?

- Yes, all of the time
- Most of the time
- Not often
- No
- Not sure
- Other

Other, please specify:

Please explain your response.

C. Infrastructure

Is there a computer /laptop/tablet available for immunization activities?

- Yes
- No

Is this computer/tablet/laptop for use exclusively for immunization program activities?

- Yes
- No

If no, what else is it used for?

Is the computer/laptop/tablet located in the immunization room?

- Yes
- No

Is there a printer available for immunization services?

- Yes
- No

Do you have internet connection?

- Yes
- No

What kind of connection do you have?

- Mobile data bundles
- Fibre/cable
- Other

Other, specify:

What type of electricity do you have?

Select all relevant

- Government Grid
- Generator only
- Solar power
- Other

Other, specify:

Does your facility experience power outages?

- Yes
- No

If yes, is there a backup electricity?

- Yes
- No

Specify backup

System Usage

How many times in the past week have you used the TImR?

- Every day
- Once
- Two – Four times
- I have not been able to use it this week because it has been broken/no electricity/no internet
- I have not been able to use it this week because someone else in my program/department has been using it
- I have not been able to use it this week because someone else in a different program/department has been using it
- I have not needed it this week
- Other

Other, please specify:

How many times in the past week have you not been able to charge the computer/laptop/tablet due to lack of electricity?

- I could charge it and use it every day.
- Once
- Two – Four times
- I have not been able to use it this week as we haven't had electricity
- Other

Other, please specify:

How many times in the past week have you not been able to send data due to lack of electricity, internet connectivity and/or data bundles?

- I could send data whenever I needed to
- Once
- Two – Four times
- I haven't been able to send data this week as we haven't had electricity/connectivity
- Other

Other, please specify:

Do you think you have enough computers/laptops/tablets for your facility?

- Yes
- No
- Not sure
- Other

Other, please specify:

In the past year, have you had any computers/laptops/tablets stop working?

- Yes, a lot
- Sometimes
- Not often
- Never
- Other

Other, please specify:

How long does it take a broken computer/laptop/tablet to get fixed or replaced?

- A day
- A week
- A month
- More than a month
- Unsure

Please describe the process to get it fixed.

Have there been any challenges that prevented you from using the TImR?

- [ ] No electricity
- [ ] No data bundle
- [ ] No internet connectivity
- [ ] Not enough computers laptops tablets
- [ ] Broken computer laptops tablets
- [ ] Other

Other, please specify:

I think the TImR is user friendly

Interviewer: This is perception (first reaction) based on the tool being simple, clear, intuitive and reliable.

- Yes
- No
- Not sure
- Other

Other, please specify:

Overall, I think that the TImR is functioning when I need to use it

- ○ Yes
- ○ No
- ○ Not sure

Other, please specify:

D.Vaccine Management

Describe how you manage vaccine stock.
Interviewer: Take note of the different tools (paper and electronic)

Has the health facility experienced any vaccine stock-out in the last 3 months?

- ○ Yes
- ○ No
- ○ Not sure

If yes, describe the reason (e.g., higher-than expected consumptions, expiry/wastage, late delivery, lack of replenishment from supplying store, other)

If yes, which vaccines were out of stock?

- ☐ Pentavalent
- ☐ MR
- ☐ Pneumococcal Conjugate Vaccine (PCV)
- ☐ HPV
- ☐ Other

Other, please specify:

What was the longest amount of time the vaccines were out of stock?

- ○ Less than 1 week
- ○ 1-2 weeks
- ○ 2-3 weeks
- ○ 1 month or more

What is your monthly requirement for Pentavelent vaccine?
Perform personal check & add the number

Is your current stock balance for Pentavalent vaccine:

- ◯ Above maximum stock (6 weeks or more)
- ◯ Appropriate stock level
- ◯ Below minimum stock (25%) (1 week or less)
- ◯ Stocked-out

Comment on the stock balances described above.

____

Does having the TImR allow you to manage stock more effectively or efficiently than before (just using the paper tools)?

- ◯ Yes,it is better using the electronic tool
- ◯ No,it was better using the paper tool
- ◯ Not sure

Explain and provide examples where possible

____

E. Immunization data

Denominators

Is a monthly target population available for the number of children under 1 year for the facility (for immunization)?

- ◯ Yes
- ◯ No

If yes, what is the source of this target population estimate?

Interviewer: This is likely the performance monitoring chart

____

What is the monthly target population of children under 1 year?

____

Do you think the target population is accurate (i.e., is it reflective of the true catchment population for this health centre)?

- ◯ Yes
- ◯ No

If no, why not?

- True catchment population is larger
- True catchment population is smaller

Does the TImR system help track individuals receiving vaccines outside their catchment areas?

- Yes
- No

If yes, please describe how

Drop-out rates

For paper-based: Calculate the drop-out rate using this formula:

*(Number of children per month vaccinated with first dose – the number of children per month vaccinated with the last dose) * 100/the number of children vaccinated with the first dose.*

For TIMR: This will be the drop-out report

What was the last months drop-out rate between Penta1 and Penta3?

What was the last months drop-out rate between MR1 and MR2?

If there has been drop-out, can you describe and provide any possible explanations for the present drop-out rates in this HF?

OUTREACH SESSIONS

Does this facility conduct outreach sessions for vaccination?

- Yes
- No

If yes, how are immunization data on outreach services managed?

- Paper based
- Electronically (TImR)
- Both paper based and electronic (TImR)

If yes, are vaccines for outreach managed through TImR?

- Yes
- No

F.Data Quality

Do you feel that the vaccine stock data quality in the TImR is:

- Much worse than the paper stock card/ledger
- A little bit worse than the paper stock card/ledger
- About the same as the paper stock card/ledger
- A little bit better than the paper stock card/ledger
- Much better than the paper stock card/ledger

Do you feel that the immunization data quality in the TImR is

Interviewer: Also consider the findings of the on-site accuracy check (if available).

- Much worse than the paper registry
- A little bit worse than the paper registry
- About the same as the paper registry
- A little bit better than the paper registry
- Much better than the paper registry

Have you had an Adverse Event Following Immunization (AEFI) reported from any of your facilities in the past year?

- Yes
- No
- Not sure

If yes, did you use the TImR to report the AEFI?

- Yes
- No
- Not sure

Do you feel that AEFI data quality in the TImR is:

- Much worse than the paper registry
- A little bit worse than the paper registry
- About the same as the paper registry
- A little bit better than the paper registry
- Much better than the paper registry
- Not applicable

If no, how do you report AEFI?

- ◯ Yes
- ◯ No
- ◯ Not sure

What are the most common challenges with getting good quality immunization and vaccine stock data? Please explain.

___

G.Reporting

Did the health facility submit all of the last 6 monthly reports?

Ask to see the last 6 months immunization monthly reports submitted by the health facility. Note: The pink form is the one that remains at the facility. The blue and yellow forms should have been submitted to the district.

- ◯ Yes, (100% of reports submitted)
- ◯ No, (<100% of reports submitted)
- ◯ Not found

Did the health facility submit all of their last 6 monthly reports on time to the district level?

- ◯ Yes, (100% of reports submitted on time)
- ◯ No, (<100% of reports submitted on time)

If no, why were they not submitted/on time?

___

Does the health facility have a system of archiving or storing paper records?

- ◯ Yes
- ◯ No
- ◯ Not sure

Do you receive feedback from the district regarding your immunization data and vaccine stock data reports?

- ◯ Yes
- ◯ No
- ◯ Not sure

Please explain

___

If yes, how often does this happen?

- Once a month
- Once a quarter
- Once a year
- Other

Please specify:

If yes, how is this feedback provided?

- Written (electronic)
- Written (paper)
- In a face-to-face meeting
- Over the phone
- Informally
- Other

Please specify:

Do you have sufficient paper-based tools for the next 3 months e.g., tally sheets, paper-based registers, barcode stickers (if relevant), child health cards, stock records etc).

- Yes
- No

If not, which tools may need to be replenished soon?

Approximately how long does/did it take to complete the receiving and put-away of vaccine supplies (for one vaccine) using paper and/or electronic forms (delivery notes and stock cards/registers/ledgers)?

Interviewer: Respondent will have to rely on their memory to estimate how long it use to take using paper forms (before the introduction of TImR).

Paper forms (where appropriate)

minutes

Electronic (where appropriate)

minutes

Since the introduction of TImR, is it:

- ◯ Faster using TImR
- ◯ Slower using TImR (it was faster using paper forms)
- ◯ No change
- ◯ Not sure

Other, please specify:

Please explain your response

Reviewing your immunization and vaccine stock data, does it indicate any issues or challenges you would like to address?

- ◯ Yes
- ◯ No
- ◯ Not sure

Other, please specify:

(Interviewer: Prompt the HW to explain the challenge and solution and see if they can relate it to the data obtained from the paper or electronic tools)

If yes, what are those challenges (on data quality)?

What would you like to do to address these challenges?

Why do you think that would work?

What prevents you from following through on the solution?

(Interviewer: If yes, ask the HW to produce the most recent data quality audit report)

Do you conduct checks on the quality/accuracy of your data (data quality audits) at your facility?

- ◯ Yes
- ◯ No
- ◯ Not sure

If yes, how often do you conduct data quality audits at your facility?

- Once a month
- Once a quarter
- Once a year
- Once every 3 – 5 years
- Not sure
- Other

Other, please specify:

Do you produce a report on the data quality audit?

- Yes
- No
- Not sure

Please explain

H. Data Analysis, Interpretation, and Use

Is there an immunization performance monitoring chart, dashboard, or other means of data visualisation (maps), available in the facility?

- Yes
- No

Please list what is available

If yes, is the performance monitoring chart, dashboard etc. up to date (updated to last complete month)?

- Yes
- No

If yes, is the chart / dashboard electronic or in paper-format?

- Electronic
- Paper
- Both

If no, how is immunization data monitored (please describe)

Is there a regular meeting to review and discuss immunization data (including vaccine stock and cold chain/temperature monitoring) in the facility among the facility staff?

- ◯ Yes
- ◯ No

If yes, is immunization service performance included in the meetings?

- ◯ Yes
- ◯ No

How is the immunization data used for decision making?

(Interviewer: Select the responses shared by the health worker and add more if necessary)

- ☐ Determining need for immunization sessions
- ☐ Determining need for outreach sessions
- ☐ Adjusting plans for outreach sessions
- ☐ Planning for staff needs
- ☐ Vaccine needs (forecasting)
- ☐ Determine training needs
- ☐ Data shared with community
- ☐ Planning and budgeting
- ☐ Other

Other, please specify:

____

Describe how decisions are made to run your immunization programme? Are you using immunization data to make decisions?

Are you able to make changes, for example, to change the amount of stock you order or receive, or to change the frequency you receive new supplies, or to transfer stock to another facility? (Interviewer: Probe for use of VIMS/EIS)

____

Please provide an example of how data from the EIS was used to make such a decision.

____

I. Workforce & Training

How many staff are working in immunization at the Health Facility, currently?

____

What was the impact of the electronic tools (TImR) on staff management (hiring of additional personnel, reorganization of responsibilities, shrinking/enlarging of immunization delivery team)?

____

J. Training and Supervision

Of the staff working in immunization, how many are trained on immunization data management activities at the facility?

____

Do you think that you are adequately trained on the electronic tools inorder to properly doyour work responsibilities?

- ○ Yes
- ○ No

Do you think that you are adequately trained on the paper based tools in order to properly do your work responsibilities?

- ○ Yes
- ○ No

If no, please explain

____

What supporting /training tools for TImR do you have for your use at the facility?

- ☐ Instruction manuals
- ☐ Job aids/quick reference sheets
- ☐ Refresher trainings
- ☐ Videos

What supporting /training tools for paper based tools do you have for your use at the facility?

- ☐ Instruction manuals
- ☐ Job aids/quick reference sheets
- ☐ Refresher trainings
- ☐ Videos

If relevant, when were these supporting/training materials(for electronic tools) last updated or reviewed?

- ○ Never
- ○ When a change was made to the system
- ○ They don't need to be updated
- ○ They need to be updated

Please Explain

____

If relevant, when were these supporting/training materials(for paper based tools) last updated or reviewed?

- ○ Never
- ○ When a change was made to the system
- ○ They don't need to be updated
- ○ They need to be updated

How many immunization training activities (including vaccine logistic management) have been conducted with the health facility in the past year?

____

If one or more activities were conducted, what type of TImR training activities have been conducted in the past year?

____

Who conducted these activities?

____

Which topics were covered?

____

Do you think that immunization staff have additional training needs?

- ○ Yes
- ○ No

If yes, what type of training?

- ☐ For conducting data collection
- ☐ For recording data
- ☐ For data analysis
- ☐ For reporting
- ☐ Other

Other, please specify

____

Do you clearly understand your role and responsibilities in using the immunization register (paper) ?

- ○ No, it is not clear
- ○ I understand my role and responsibilities but I am not properly equipped
- ○ I understand my role and responsibilities, I am well equipped to carry out my responsibilities

Do you clearly understand your role and responsibilities in using TImR?

- No, it is not clear and there is no standard operating procedure (SoP)
- I understand my role and responsibilities but there is no SoP at this time
- I understand my role and responsibilities and there is a SoP
- I understand my role and responsibilities, there is a SoP and I am well trained to carry out my responsibility
- There is a clear understanding of all roles and responsibilities and an SoP is kept up to date

If there is a problem with the TImR, do you have timely access to support from the district/region or from other places

- Yes
- No
- Not sure

Please explain

____

How many immunization supervision activities has the district or higher level conducted with the health facility in the past year?

- No supervision
- Once least once a year
- Once a quarter
- Once a month
- Other

Other, please specify

____

If one or more immunization supervision activities were conducted, did you use the TImR or data from the TImR to guide your discussion?

- Yes
- No

If yes, please describe how the data was used?

____

K. Close and Comments

If another health facility was considering using the TImR for their immunization records and vaccine logistics management and asked you for advice, what would you tell them about benefits, challenges and best practices?

____

Do you think an electronic tool (to manage vaccine stock and immunization data) does/would make your job easier?

- ◯ Yes
- ◯ No
- ◯ Not sure

Please describe how it could/does improve your work?

If relevant, do you have any advice on how to improve TImR?

What electronic tools are you using to manage COVID-19 vaccine delivery e.g. for stock management and/or for individual vaccination

---

Final notes and comments

K. Final notes and comments (of the interviewer)

Please summarise your observations of the day, focusing on the "WHY?". Why is TImR being used, or not being used? Why does the HW like, or not, like the tool?
This is for the Team Lead to summarise the key findings

Is there anything important that has not been captured in the interview guide?
