# Supplementary material for: Impact of electronic immunization registries and electronic logistics management information systems in four low-and middle-income countries: Guinea, Honduras, Rwanda, and Tanzania
Source: Vaccine. 2025 Apr 30;54:None. doi: 10.1016/j.vaccine.2025.127066 (PMC12132044; doi:10.1016/j.vaccine.2025.127066)
Supplement: Supplementary file 5 — Supplementary material 5 [file mmc5.pdf]

On-site accuracy check (for all facilities)

On-site accuracy check (for all facilities)

Date of interview

yyyy-mm-dd

Name of interviewer

Province / Region

- ◯ Mbeya
- ◯ Njombe
- ◯ Tanga
- ◯ Kilimanjaro
- ◯ Arusha
- ◯ Dodoma
- ◯ Singida
- ◯ Shinyanga
- ◯ Mwanza
- ◯ Pwani

District

Health facility name

Is this the first or second health facility visited in the district?

- ◯ First facility visited
- ◯ Second facility visited

Role within immunization service

1

* **Name of interviewee**

Role within immunization services

- ◯ Head of clinic
- ◯ Vaccinator
- ◯ Data capture
- ◯ Other

* **others specify**

enter only when others is selected

System in use

Is TImR available at the facility?

- ◯ Yes
- ◯ No

Instructions & child

1

» Entries per child

If present during the vaccination sessions, select three children vaccinated. If not, and if using TImR, then find the last three (3) entries for new registrations in TImR. Match these entries to the equivalent entries in the Child Register (Book 7). Select (yes) if entries match, (no) is entries do not match. The district immunization officer can assist the data collector in conducting this assessment.

Child's name in Child Health/RCH1 card

- ◯ Yes
- ◯ No

Child's name in Child Register (Book 7)

- ◯ Yes
- ◯ No

Child's name in TImR

- ◯ Yes
- ◯ No

TImR number in Child Health/RCH1 card

- ◯ Yes
- ◯ No

TImR number in Child Register (Book 7)

- ◯ Yes
- ◯ No

TImR number in TImR

- ◯ Yes
- ◯ No

Child's date of birth in Child Health/RCH1 card

- Yes
- No

Child's date of birth in Child Register (Book 7)

- Yes
- No

Child's date of birth in TImR

- Yes
- No

Sex (m/f) in Child Health/RCH1 card

- Yes
- No

Sex (m/f) in Child Register (Book 7)

- Yes
- No

Sex (m/f) in TImR

- Yes
- No

Penta in Child Health/RCH1 card

- Yes
- No

Penta in Child Register (Book 7)

- Yes
- No

Penta in TImR

- Yes
- No

MR in Child Health/RCH1 card

- Yes
- No

MR in Child Register (Book 7)

- ○ Yes
- ○ No

MR in TImR

- ○ Yes
- ○ No

PCV in Child Health/RCH1 card

- ○ Yes
- ○ No

PCV in Child Register (Book 7)

- ○ Yes
- ○ No

PCV in TImR

- ○ Yes
- ○ No

Summary

How do the matched entries compare?

- ○ All of them exactly the same
- ○ There are some differences
- ○ Matched entries are mostly different

How does the health worker (HW) explain the differences?

____

Which record does the HW believe to be most accurate?

- ○ TImR (where applicable)
- ○ Child Health/RCH1 card
- ○ Paper registry
- ○ Not sure

Why it is more accurate?

____

What are the most common differences/errors between the records? Please explain.

____

When are the vaccinations recorded in the paper tools?

- ◯ Before vaccination administered
- ◯ During vaccination session
- ◯ Immediately after vaccination administered
- ◯ Long after vaccination administered
- ◯ Tools not used during vaccination session
- ◯ Other

Please specify

____

When are the vaccinations recorded in the TImR (where relevant)?

- ◯ Before vaccination administered
- ◯ During vaccination session
- ◯ Immediately after vaccination administered
- ◯ Long after vaccination administered
- ◯ Tools not used during vaccination session
- ◯ Other

Please specify

____

Observations/comments (of the interviewer)

____
