# Supplementary material for: Impact of electronic immunization registries and electronic logistics management information systems in four low-and middle-income countries: Guinea, Honduras, Rwanda, and Tanzania
Source: Vaccine. 2025 Apr 30;54:None. doi: 10.1016/j.vaccine.2025.127066 (PMC12132044; doi:10.1016/j.vaccine.2025.127066)
Supplement: Supplementary file 6 — Supplementary material 6 [file mmc6.pdf]

FACILITY SURVEY (only for facilities where TImR exists)

FACILITY SURVEY (only for facilities where TImR exists)

Is TImR available at the facility?

- ◯ Yes
- ◯ No

This survey is not required (only for facilities where TImR exists).

FACILITY SURVEY (only for facilities where TImR exists)

Date of interview

yyyy-mm-dd

Name of interviewer

Province / Region

- ◯ Mbeya
- ◯ Njombe
- ◯ Tanga
- ◯ Kilimanjaro
- ◯ Arusha
- ◯ Dodoma
- ◯ Singida
- ◯ Shinyanga
- ◯ Mwanza
- ◯ Pwani

District

Health facility name

Is this the first or second health facility visited in the district?

- ◯ First facility visited
- ◯ Second facility visited

Role within immunization service

1

* **Name of interviewee**

Role within immunization services

- Head of clinic
- Vaccinator
- Data capturer
- Other

Others, specify

Enter only when others is selected

A. User experience & training

1. Which of the following describes your training with the TImR

- I have not been trained on the TImR
- I have been trained on the TImR and the training was adequate to my needs
- I have been trained on the TImR and the training was not adequate to my needs

2. Which of the following describes your work experience with the TImR

- I have not yet used the TImR in my work
- I use the TImR in my work occasionally
- I use the TImR in my work regularly
- I previously used the TImR in my work, but I no longer use the TImR

3. How long has your workplace been using the TImR?

- TImR is not used in my workplace
- Less than 1 month
- 1 – 6 months
- More than 6 months

4. Does your workplace use computers/tablets/smart phones etc for any clinical service or stock management except immunization?

- Yes
- No

If yes, what?

5. Are vaccinations administered in outreach and mobile services recorded in the TImR?

- Always
- Usually
- Rarely
- Never
- Only partially (for stock tracking but not for individuals vaccinated)

6. Which of the following best describes your work:

- My work is administrative, or in data management. I rarely or never provide clinical care.
- I work mostly with vaccination delivery
- I work mostly with the vaccines and cold chain management

B. Computer literacy

---

1. I am interested in working with computers/tablets/smartphone

- Yes
- No

2. I have moderate (or greater) skill in using computers/tablets/smartphone

- Yes
- No

3. I feel that using computers/tablets/smartphone will support me to be more efficient at work

- Yes
- No

C. Infrastructure

1.My facility or office has a connection to the internet that is adequate for us to effectively use the TImR

- Yes
- No

2.My facility or office has consistent access to electricity that is adequate for us to effectively use the TImR

- Yes
- No

3. I can access computers/tablets/smartphone in my workplace when I need them to use the TImR

- Yes
- No

D. Information Quality

The TImR provides sufficient information to enable me to do my tasks

- Yes
- No

I am satisfied with the accuracy and completeness of the vaccine stock and immunization records in the TImR

- Yes
- No

With the TImR, I am able to access the information I need, when I need it

- Yes
- No

The TImR is in a format that quickly gives me the information I need

- Yes
- No

E. Service Quality

The TImR is dependable

- Yes
- No

The phone/tablet I use is usable (not damaged or disabled) and easy to use

- Yes
- No

My supervisor has been helpful in supporting my use of the TImR

- Yes
- No

The IT support for issues with the TImR is timely (e.g., assistance logging in)

- Yes
- No

The reported bugs (problems) in the software get fixed in an acceptable time frame

- Yes
- No

(if applicable) The user guides or help functions in the TImR are useful

- Yes
- No

F. Use

I frequently use the TImR for my tasks

- Yes
- No

I am dependent on the TImR for at least one of my assigned tasks

- Yes
- No

Our facility regularly uses TImR to generate our monthly reports

- Yes
- No

Our facility regularly uses the TImR to generate a list of defaulters

- Yes
- No

Our facility regularly uses the TImR to generate recall or reminder messages for parents

- Yes
- No

Our facility regularly uses the TImR to generate new records of immunization for children that have lost their child health card

- Yes
- No

Our facility regularly uses the TImR to record stock transactions (receipts, issues, wastage)

- Yes
- No

Our facility regularly uses TImR to identify near-to-expiry vaccines

- Yes
- No

Our facility regularly uses TImR to order new supplies

- Yes
- No

Our facility regularly and correctly monitors cold chain equipment (refrigerator) temperature using remote temperature monitors that send data to VIMS

- Yes
- No

G. User Satisfaction / Perceived Benefit

Overall, I am satisfied with the TImR

- Yes
- No

The TImR is easy to use

- Yes
- No

I can finish my tasks faster by using the TImR

- Yes
- No

The TImR improves my productivity / makes me more effective

- Yes
- No

The TImR has a positive impact on the quality of my work

- Yes
- No

I am confident that the TImR makes immunization services better

- Yes
- No

I trust that the data in the TImR will not be lost

- Yes
- No

H.Caregiver satisfaction

I think that caregiver's satisfaction has improved since we have started using the TImR

- Yes
- No

I.Comments

Do you have any other comments or experiences that we have not asked about, or more information regarding any of the questions above that you wish to share? Can you give an example of how the TImR has impacted your work (for better or for worse)?
