# Supplementary material for: Impact of electronic immunization registries and electronic logistics management information systems in four low-and middle-income countries: Guinea, Honduras, Rwanda, and Tanzania
Source: Vaccine. 2025 Apr 30;54:None. doi: 10.1016/j.vaccine.2025.127066 (PMC12132044; doi:10.1016/j.vaccine.2025.127066)
Supplement: Supplementary file 7 — Supplementary material 7 [file mmc7.docx]

## SUPPLEMENTARY DATA (ANNEX)


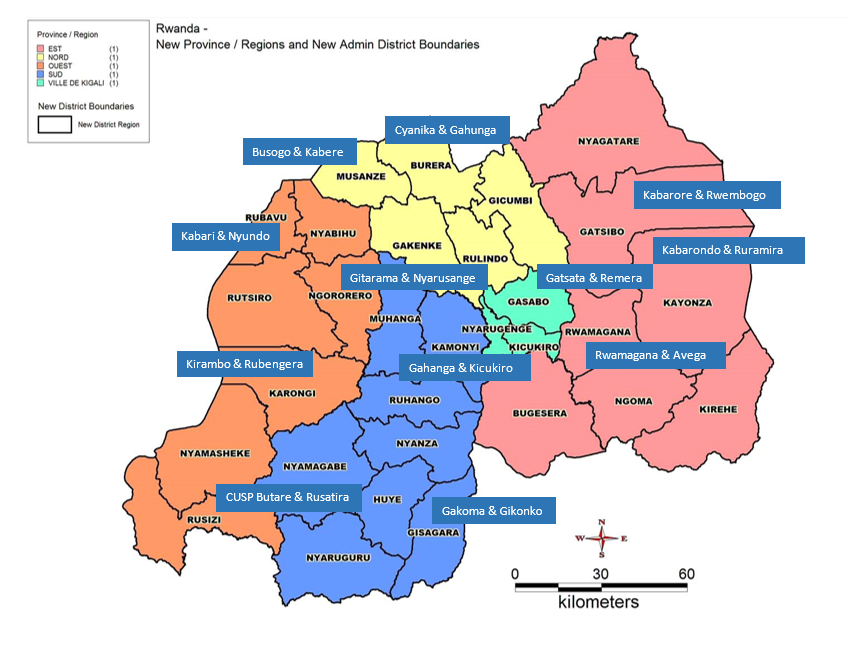


Figure 8: Rwanda - Mapping of health facilities in the final sample

Figure 9: Tanzania - Purposive sampling of regions

Figure 10: Tanzania - Sample composition depicting 3 sample selection stages from region to health facility level*. [HF = health facility]*


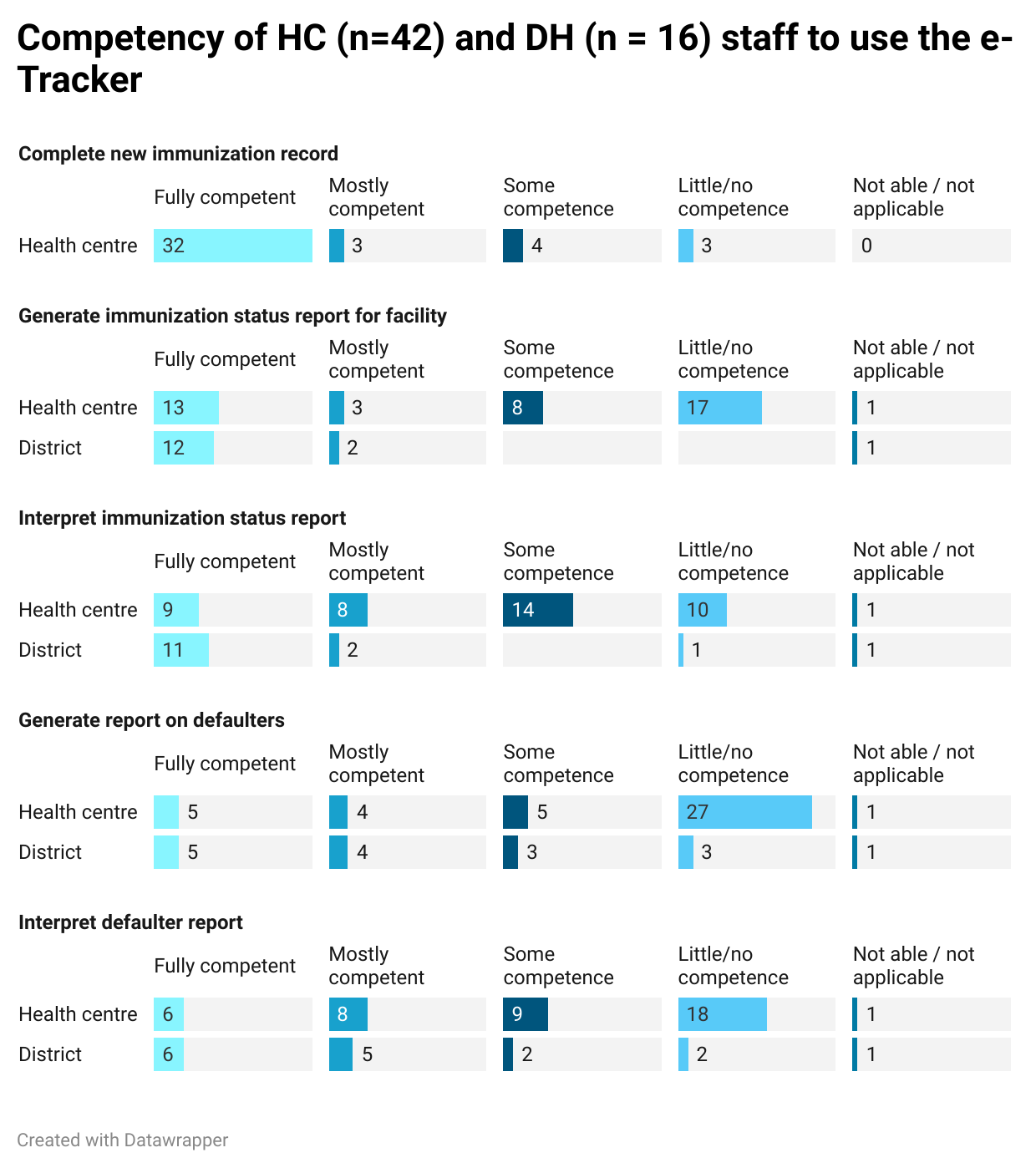


Figure 11: Competency of Health Centre (n = 42) and District Hospital (n = 16) staff to use the e-Tracker


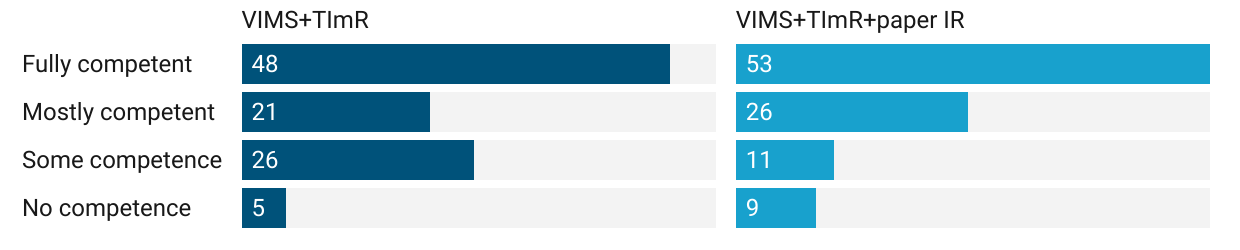


Figure 12: Tanzania - Competency of HF users using the tool (%) *[VIMS = Vaccine Information Management System; TImR = Tanzania Immunization Registry; IR = Immunization Register]*


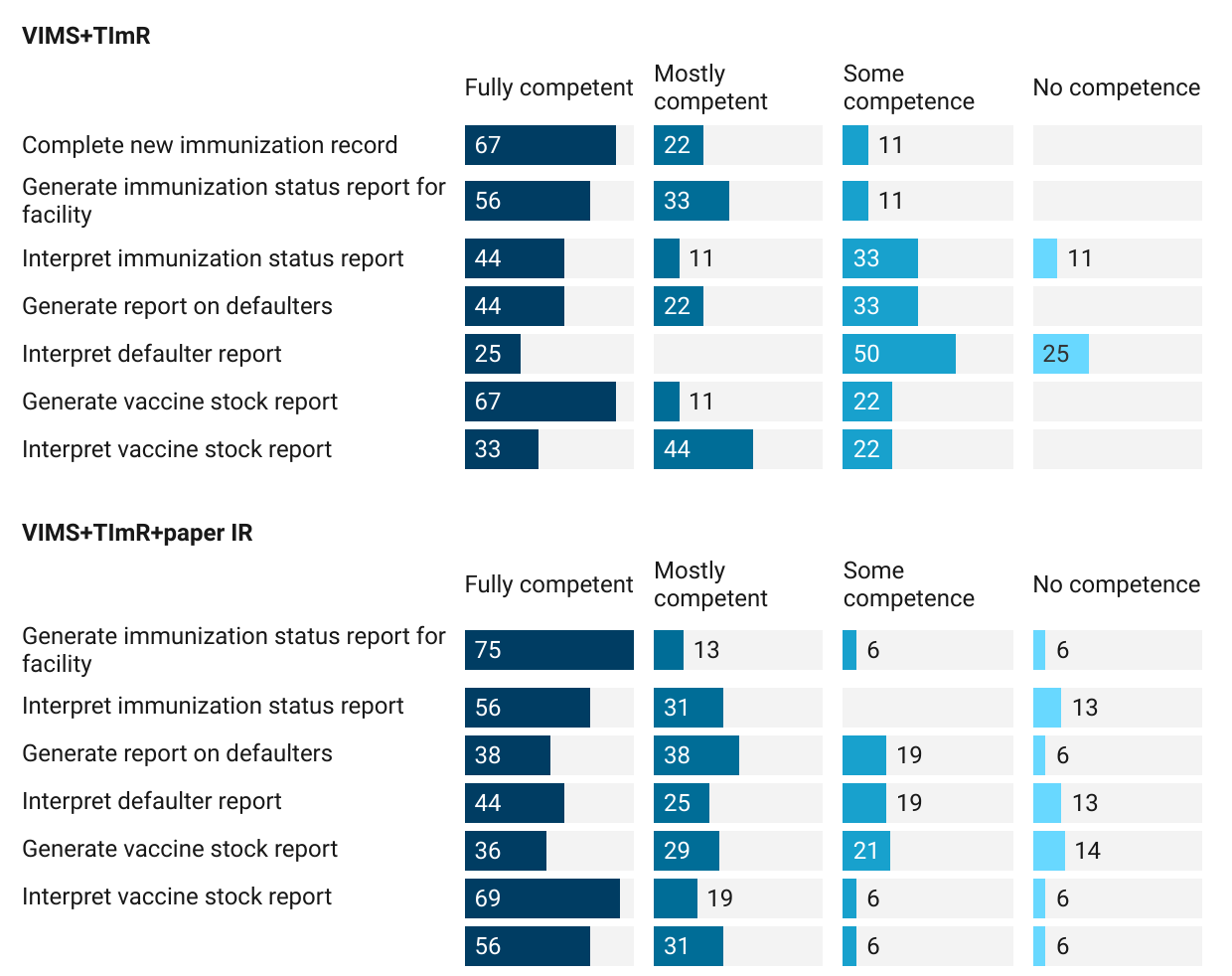


Figure 13: Competency of VIMS+TImR (n=10) and VIMS+TImR+paper IR users for identified activities (%) *[VIMS = Vaccine Information Management System; TImR = Tanzania Immunization Registry; IR = Immunization Register]*


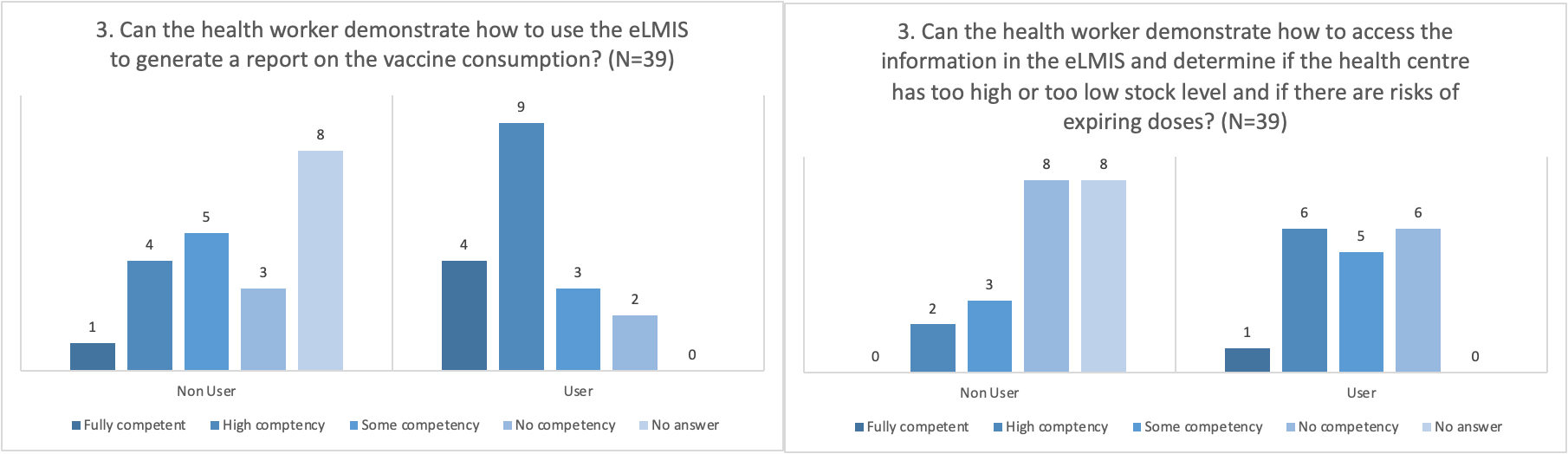


Figure 14: Level of competence of health workers in using eLMIS. *[eLMIS = electronic logistic management information system]*
