# Supplementary material for: Impact of electronic immunization registries and electronic logistics management information systems in four low-and middle-income countries: Guinea, Honduras, Rwanda, and Tanzania
Source: Vaccine. 2025 Apr 30;54:None. doi: 10.1016/j.vaccine.2025.127066 (PMC12132044; doi:10.1016/j.vaccine.2025.127066)
Supplement: Supplementary file 8 — Supplementary material 8 [file mmc8.docx]

## SUPPLEMENTARY DATA (Annex)

| **Country** | **WHO region** | **Income level** | **eIR** | **eIR: Pilot** | **eLMIS** | **eLMIS: Pilot** | **Reason for exclusion (otherwise, eligible)** |
| --- | --- | --- | --- | --- | --- | --- | --- |
| Afghanistan | EMRO | LIC | Yes | No | No | 0 | Eligible eIR (no eLMIS) |
| Albania | EURO | UMIC | Yes | Yes | 0 | 0 | Income level |
| Anguilla | PAHO | HIC | Yes | Yes | No | 0 | Income level |
| Argentina | PAHO | UMIC | Yes | No | No | 0 | Income level |
| Bahamas | PAHO | HIC | Yes | Yes | No | 0 | Income level |
| Bangladesh | SEARO | LMIC | Yes | No | Yes | No | Eligible eIR and eLMIS (DHIS2 model use as both) |
| Belize | PAHO | UMIC | No | 0 | No | 0 | Income level |
| Benin | AFRO | LIC | Yes | Yes | yes | No | Eligible eIR and eLMIS (insufficient scale) |
| Bermuda | PAHO | HIC | Yes | Yes | No | 0 | Income level |
| Bolivia | PAHO | LMIC | Yes | Yes | No | 0 | Insufficient scale (eIR in pilot / no eLMIS) |
| Brazil | PAHO | UMIC | Yes | No | Yes | Yes | Income level |
| British Virgin Islands | PAHO | HIC | Yes | Yes | No | 0 | Income level |
| Chile | PAHO | HIC | Yes | No | No | 0 | Income level |
| China | WPRO | UMIC | Yes | Yes | 0 | 0 | Income level |
| Colombia | PAHO | UMIC | Yes | No | Yes | Yes | Eligible eIR (no eLMIS) -exception from Income level exclusion |
| Costa Rica | PAHO | UMIC | Yes | 0 | No | 0 | Income level |
| Ecuador | PAHO | UMIC | Yes | 0 | No | 0 | Income level |
| Gambia | AFRO | LIC | Yes | No | Yes | No | Eligible eIR and eLMIS |
| Georgia | EURO | UMIC | Yes | No | 0 | 0 | Income level |
| Ghana | AFRO | LMIC | Yes | No | Yes | No | Eligible eIR and eLMIS |
| Grenada | PAHO | UMIC | Yes | No | No | 0 | Income level |
| Guatemala | PAHO | UMIC | Yes | No | No | 0 | Income level |
| Guinea | AFRO | LIC | No | 0 | Yes | No | Eligible eLMIS (no eIR) |
| Honduras | PAHO | LMIC | Yes | No | No | No | Eligible eIR (no eLMIS) |
| India | SEARO | LMIC | Yes | No | Yes | No | Eligible eIR and eLMIS |
| Indonesia | SEARO | UMIC | Yes | Yes | Yes | Yes | Eligible eLMIS (insufficient scale of eLMIS) - exception from Income level exclusion |
| Ivory Coast | AFRO | LMIC | Yes | No | No | 0 | Eligible eIR (no eLMIS) |
| Jamaica | PAHO | UMIC | No | 0 | No | 0 | Income level |
| Jordan | EMRO | UMIC | Yes | 0 | 0 | 0 | Income level |
| Kenya | AFRO | LMIC | Yes | Yes | Yes | No | Eligible eLMIS (insufficient scale of eIR) |
| Malawi | AFRO | LIC | Yes | Yes | Yes | No | Eligible eLMIS (insufficient scale of eIR) |
| Mexico | PAHO | UMIC | Yes | No | No | 0 | Income level |
| Montserrat | PAHO | UMIC | Yes | Yes | No | 0 | Income level |
| Mozambique | AFRO | LIC | Yes | Yes | Yes | No | Eligible eLMIS (insufficient scale of eIR) |
| Myanmar | SEARO | LMIC | No | 0 | Yes | Yes | Insufficient scale (no eIR / eLMIS in pilot) |
| Nepal | SEARO | LMIC | Yes | Yes | Yes | Yes | Insufficient scale (eIR and eLMIS in pilot) |
| Pakistan | EMRO | LMIC | Yes | No | Yes | No | Eligible eIR and eLMIS |
| Panama | PAHO | HIC | Yes | No | No | 0 | Income level |
| Paraguay | PAHO | UMIC | Yes | No | No | 0 | Income level |
| Peru | PAHO | UMIC | Yes | No | No | 0 | Income level |
| Republica Dominicana | PAHO | UMIC | No | 0 | No | 0 | Income level |
| Rwanda | AFRO | LIC | Yes | No | Yes | No | Eligible eIR and eLMIS |
| Senegal | AFRO | LIC | Yes | No | No | Yes | Eligible eIR (insufficient scale of eLMIS) |
| Sri Lanka | SEARO | LMIC | Yes | Yes | No | No | Insufficient scale (eIR in pilot / eLMIS discontinued) |
| St Kitts and Nevis | PAHO | HIC | Yes | No | No | 0 | Income level |
| Suriname | PAHO | UMIC | No | 0 | No | 0 | Income level |
| Tanzania | AFRO | LIC | Yes | No | Yes | No | Eligible eIR and eLMIS |
| Thailand | SEARO | UMIC | Yes | Yes | No | 0 | Income level |
| Turks and Caicos | PAHO | HIC | Yes | Yes | No | 0 | Income level |
| Uganda | AFRO | LIC | Yes | No | Yes | Yes | Eligible eIR (insufficient scale of eLMIS) |
| Uruguay | PAHO | HIC | Yes | No | No | 0 | Income level |
| Venezuela | PAHO | UMIC | Yes | Yes | No | 0 | Income level |
| Vietnam | WPRO | LMIC | Yes | No | Yes | No | Eligible eIR and eLMIS |
| Zambia | AFRO | LMIC | Yes | No | Yes | No | Eligible eIR and eLMIS |

Table 3: List of potential low- and middle-income countries with eIR and/or eLMIS. *[“0” denotes missing information]*

| Region | # of HC with eLMIS | # of HC without eLMIS | Total # of HCs (% with eLMIS) | # of districts per region | Target Population, 2021** | Total population per region, 2021* | Penta3 Coverage, 2020*** |
| --- | --- | --- | --- | --- | --- | --- | --- |
| *Boké* | *14* | *28* | *42 (50%)* | *5* | *47,882* | *1,330,079* | *91%* |
| *Conakry* | *19* | *24* | *43 (83%)* | *5* | *73,428* | *2,039,725* | *77%* |
| *Kindia* | *15* | *43* | *58 (35%)* | *5* | *56,407* | *1,916,276* | *100%* |
| Labé | 11 | 47 | 58 (12%) | 5 | 43,898 | 1,219,391 | 77% |
| Faranah | 0 | 49 | 49 (0%) | 4 | 41,625 | 1,156,311 | 88% |
| Kankan | 0 | 69 | 69 (0%) | 5 | 86,754 | 2,409,867 | 97% |
| *Mamou* | *0* | *41* | *41 (0%)* | *3* | *32,309* | *897,518* | *93%* |
| *N'Zérékoré* | *0* | *84* | *84 (0%)* | *6* | *77,529* | *1,330,079* | *103%* |
| Total | 59 | 385 | 444 (13%) | 38 | 459,832 | 12,907,394 | 91% |

Table 4: Guinea - eLMIS distribution across health centers, number of districts, target population in 2021 and Penta 3 coverage in 2020 per region. Selected regions are shadowed and in italics. *[HC = Health Centre; eLMIS = electronic logistics management information system; Penta3 = third dose of pentavalent vaccine]*

|  | District | # of HC with eLMIS | # of HC without eLMIS | Total # of HCs (% with eLMIS) | Target Population, 2021 | Total Population per District, 2021 | Penta3 Coverage, 2020 |
| --- | --- | --- | --- | --- | --- | --- | --- |
| Boké | *Boffa* | *2* | *6* | *8 (25%)* | *10,443* | *261,085* | *85%* |
|  | *Boké* | *5* | *8* | *13 (38%)* | *22,108* | *552,704* | *93%* |
|  | Fria | 3 | 3 | 6 (50%) | 5,015 | 125,376 | 93% |
|  | Gaoual | 2 | 6 | 8 (25%) | 4,751 | 118,795 | 92% |
|  | Koundara | 2 | 5 | 7 (29%) | 6,384 | 159,602 | 91% |
| Conakry | Dixinn | 2 | 2 | 4 (50%) | 6,006 | 135,788 | 86% |
|  | Kaloum | 4 | 1 | 5 (80%) | 2,763 | 62,457 | 91% |
|  | *Matam* | *2* | *2* | *4 (50%)* | *6,337* | *143,255* | *64%* |
|  | Matoto | 3 | 8 | 11 (27%) | 29,460 | 665,908 | 58% |
|  | *Ratoma* | *8* | *11* | *19 (42%)* | *28,862* | *652,406* | *84%* |
| Kindia | Coyah | 3 | 3 | 6 (50%) | 13,667 | 341,689 | 102% |
|  | *Dubréka* | *4* | *8* | *12 (33%)* | *17,116* | *427,915* | *96%* |
|  | Forécariah | 2 | 9 | 11 (18%) | 12,591 | 314,786 | 110% |
|  | Kindia | 5 | 10 | 15 (33%) | 22,772 | 569,301 | 91% |
|  | Télimelé | 1 | 13 | 14 (7%) | 14,749 | 368,737 | 86% |
| Mamou | Dalaba | 0 | 10 | 10 (0%) | 6,930 | 173,272 | 100% |
|  | *Mamou* | *0* | *18* | *18 (0%)* | *16,539* | *413,480* | *71%* |
|  | Pita | 0 | 13 | 13 (0%) | 14.419 | 360,484 | 109% |
| N'Zérékoré | Beyla | 0 | 16 | 16 (0%) | 16,917 | 422,927 | 98% |
|  | Guéckédou | 0 | 13 | 13 (0%) | 15,066 | 376,670 | 97% |
|  | Lola | 0 | 9 | 9 (0%) | 8,900 | 222,512 | 92% |
|  | Macenta | 0 | 18 | 18 (0%) | 14,444 | 361,119 | 114% |
|  | Nzérékoré | 0 | 18 | 18 (0%) | 20,565 | 514,129 | 104% |
|  | *Yomou* | *0* | *10* | *10 (0%)* | *5,929* | *148,238* | *114%* |
| *Total* | | **48** | **220** | **268 (18%)** | **322,733** | **7,892,635** | **93%** |

Table 5: Guinea - eLMIS distribution across health centers, target population in 2021 and Penta 3 coverage in 2020 per district in the 5 selected regions. Selected districts are shadowed and in italics. *[HC = Health Centre; eLMIS = electronic logistics management information system; Penta3 = third dose of pentavalent vaccine]*

|  | | **Selected Districts (n=84)** | | **Sample (n=42)** | |
| --- | --- | --- | --- | --- | --- |
| **Criteria** | **Detail** | **With eLMIS** | **No eLMIS** | **With eLMIS** | **No eLMIS** |
| **Type of health facility** | Communal Medical Centers (CMC) | 5 (6%) | 4 (5%) | 5 (12%) | 0 (0%) |
|  | Health centers (HC) | 16 (19%) | 59 (70%) | 15 (36%) | 22 (52%) |
| **Location** | Rural | 2 (2%) | 39 (46%) | 2 (5%) | 12 (29%) |
|  | Urban | 19 (23%) | 24 (29%) | 18 (43%) | 10 (24%) |
| **Total** | | **21 (25%)** | **63 (75%)** | **20 (48%)** | **22 (52%)** |

Table 6: Guinea - Characteristics of the health centers: sample vs. distribution in selected districts *[eLMIS = electronic logistics management information system]*

|  | | | | | |  |  |  |  |  |  |
| --- | --- | --- | --- | --- | --- | --- | --- | --- | --- | --- | --- |
| **Registro Único de Prestadores de Servicios de Salud (RUPS) 2022** | | | | |  | | **Sample** | | | |  |
|  | **Hospital Policlinico** | **CIS** | **UAPS** | **Grand Total** |  | | **Hospital Policlinico** | **CIS** | **UAPS** | **Grand Total** |  |
| Atlántida | 14 | 17 | 33 | **64** |  | | 2 | 4 | 4 | 10 |  |
| Choluteca | 3 | 28 | 67 | **98** |  | |  |  |  |  |  |
| Colón | 2 | 22 | 26 | **50** |  | |  |  |  |  |  |
| Comayagua | 3 | 35 | 48 | **86** |  | | 0 | 6 | 4 | **10** |  |
| Copán | 3 | 22 | 67 | **92** |  | | 1 | 5 | 4 | **10** |  |
| Cortés | 12 | 51 | 69 | **132** |  | |  |  |  |  |  |
| El Paraíso | 2 | 31 | 58 | **91** |  | | 1 | 7 | 2 | **10** |  |
| Francisco Morazán | 18 | 56 | 115 | **189** |  | | 0 | 5 | 5 | **10** |  |
| Gracias a Dios | 6 | 10 | 21 | **37** |  | |  |  |  |  |  |
| Intibucá | 1 | 21 | 33 | **55** |  | | 0 | 5 | 5 | **10** |  |
| Islas de la Bahía | 1 | 4 | 7 | **12** |  | |  |  |  |  |  |
| La Paz | 1 | 22 | 25 | **48** |  | | 1 | 5 | 4 | **10** |  |
| Lempira | 1 | 35 | 79 | **115** |  | |  |  |  |  |  |
| Ocotepeque | 1 | 10 | 28 | **39** |  | |  |  |  |  |  |
| Olancho | 6 | 35 | 56 | **97** |  | |  |  |  |  |  |
| Santa Bárbara | 3 | 25 | 53 | **81** |  | | 0 | 6 | 4 | **10** |  |
| Valle | 2 | 22 | 15 | **39** |  | |  |  |  |  |  |
| Yoro | 8 | 31 | 54 | **93** |  | |  |  |  |  |  |
| Grand Total | **87** | **477** | **854** | **1418** |  | | **5** | **43** | **32** | **80** |  |
| % in selected regions | **6%** | **32%** | **61%** | **100%** |  | | **6%** | **54%** | **40%** | **100%** |  |
| % in the country | **6%** | **34%** | **60%** | **100%** |  | |  |  |  |  |  |

Table 7: Honduras - Health facility types in the sample compared to the distribution in the country *[CIS = Centro Integral de Salud; UAPS = Unidad de Atención Primaria de Salud]*

| **Criteria** | **Detail** | **National (n=505)** | **Sample (n=24)** |
| --- | --- | --- | --- |
| **Type of health facility** | FBO/NGO | 28% | 46% |
|  | Public | 68% | 54% |
| **Size of catchment population <1yr** | High | 46% | 79% |
|  | Low | 49% | 21% |
| **Penta3 drop-out** | High | 7% | 21% |
|  | Low | 49% | 67% |
|  | None | 40% | 13% |
| **MR drop-out** | High | 8% | 8% |
|  | Low | 28% | 33% |
| **Location** | None | 59% | 58% |
|  | Rural | 86% | 58% |
|  | Urban | 9% | 42% |
| **HMIS reported data** |  | 30% | 28% |

Table 8: Rwanda - Characteristics of the health centers: sample vs distribution in selected districts.

| **Regions** | **VIMS+TImR*** | **VIMS+paper IR** |
| --- | --- | --- |
| **Sample** | Dodoma | Singida |
|  | Mwanza (transitioning to fully electronic) | Shinyanga |
|  | Njombe | Mbeya |
|  | Kilimanjaro (transitioning to fully electronic) | Pwani |
|  | Arusha |  |
|  | Tanga (fully electronic) |  |
| **Total in sample (n=10)** | **6 (60%)** | **4 (40%)** |
| **Total in country (n=26)** | **15 (58%)** | **11 (42%)** |

Table 9: Tanzania - Regional pairs included in the evaluation and comparison with distribution of tool use in country. *[VIMS = Vaccine Information Management System; TImR = Tanzania Immunization Registry; IR = Immunization Register]*

* All regions expected to operate a parallel electronic/ paper system, unless otherwise specified

|  | Description | Distribution across selected regions | | | Distribution in sample | | |
| --- | --- | --- | --- | --- | --- | --- | --- |
|  |  | VIMS+TImR | VIMS+paper IR | **Total** | VIMS+TImR | VIMS+paper IR | **Total** |
| Location / District type | Rural: Town Council (TC) or District Council (DC) | 73% | 77% | **74%** | 70% | 75% | **72%** |
|  | Urban: Municipal Council (MC) or City Council (CC) | 27% | 23% | **26%** | 30% | 25% | **28%** |
| HF Type | Dispensary | 80% | 80% | **80%** | 76% | 75% | **76%** |
|  | Health Center | 14% | 12% | **13%** | 16% | 17% | **16%** |
|  | Hospital | 4% | 5% | **5%** | 8% | 8% | **8%** |
|  | Other privately owned facilities (clinics, maternity homes, laboratories, etc.) | 2% | 3% | **2%** | 0% | 0% | **0%** |

Table 10: Tanzania - Distribution of characteristics of health facilities in the ten regions and in the sample *[VIMS = Vaccine Information Management System; TImR = Tanzania Immunization Registry; IR = Immunization Register]*
